# GPR160 is a potential biomarker associated with prostate cancer

**DOI:** 10.1038/s41392-021-00583-7

**Published:** 2021-06-25

**Authors:** Wanjing Guo, Junyu Zhang, Yan Zhou, Caihong Zhou, Yunjie Yang, Zhaotong Cong, Jibin Dong, Dehua Yang, Bo Dai, Ming-Wei Wang

**Affiliations:** 1 The National Center for Drug Screening and CAS Key Laboratory of Receptor Research, Shanghai Institute of Materia Medica, Chinese Academy of Sciences (CAS), Shanghai, China; 2 University of Chinese Academy of Sciences, Beijing, China; 3 Department of Urology, Fudan University Shanghai Cancer Center, Shanghai, China; 4 School of Pharmacy, Fudan University, Shanghai, China

**Keywords:** Tumour biomarkers, Urological cancer

**Dear Editor**,

Prostate cancer (PC) is one of the most common noncutaneous cancers among men worldwide with a relatively higher incidence and mortality rate.^1^ Conventional screening for prostate cancer relies on prostate-specific antigen (PSA), a valuable biomarker but has some deficiencies. Radical prostatectomy, androgen ablation, and radiotherapy are still commonly used treatments for localized prostate cancer. Androgen ablation is a procedure aimed at suppressing hormone production or blocking the function of the androgen receptor. At the early stage, prostate cancer growth is dependent on androgen, while most patients will ultimately transform to a hormone-refractory state. New diagnostic tools and therapeutic strategies are thus in high demand to curtail this situation. GPR160 belongs to the class A GPCR subfamily and was de-orphanized recently. Its endogenous ligand is CARTp (cocaine- and amphetamine-regulated transcript peptide) responsible for neuropathic pain in rodents.^2^ We have shown previously that GPR160 is highly expressed in prostate cancer tissue samples and its knockdown by lentivirus-mediated short hairpin RNA constructs targeting the human *GPR160* gene (ShGPR160) resulted in prostate cancer cell apoptosis and growth arrest both in vitro and in athymic mice.^3^

To investigate the relationship between expression of GPR160 and prostate cancer clinicopathologic characteristics, we examined the expression of GPR160 in prostate samples obtained from 224 patients during prostatectomy by in situ hybridization and immunohistochemistry (Figure S1). Of the tissue samples obtained from 224 patients receiving prostatectomy, 199 and 158 of them were successfully assessed for RNAscope® and immunohistochemistry analyses, respectively (Fig. 1a). Positive readouts for GPR160 expression were 77.4% (154/199) and 65.8% (104/158) in mRNA and protein levels, respectively; both were significantly higher in cancerous than that in adjacent normal tissues (*P* < 0.0001, Figs. 1b–e), demonstrating consistency between the two parameters.

**Fig. 1:**
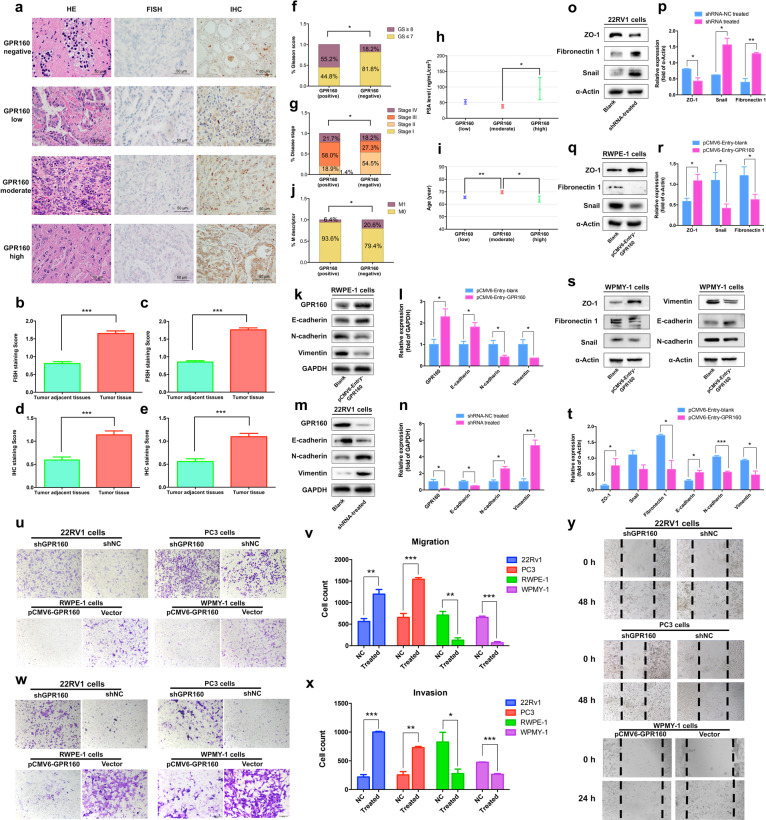
Higher expression of GPR160 in cancerous human prostate tissues and relationship between GPR160 expression levels and clinical characteristics of enrolled patients. **a** Images of GPR160 expression detected by RNAscope^®^ (mRNA, FISH)) and immunohistochemistry (protein, IHC). HE, hematoxylin and eosin, scale bars, 50 μm. **b** 143 samples containing both normal and cancerous tissues used for mRNA analysis with paired *t* test. **c** 167 samples containing cancerous and 143 samples containing normal tissues used for mRNA analysis with unpaired *t* test. **d** 112 samples containing both normal and cancerous tissues used for immunohistochemistry analysis with paired *t* test. **e** 140 samples containing cancerous and 112 samples containing normal tissues used analysis with unpaired *t* test. Data presented are means ± SEM of up to three independent reading; ***P < 0.0001. **f** Gleason scores vs. GPR160 mRNA levels in the prostate cancer tissue. **g** Disease stage vs. GPR160 mRNA levels in the prostate cancer tissue. **h** PSA levels in patients with elevated GPR160 mRNA transcription. **i** Age-related GPR160 protein intensity. **j** M scores in patients with or without GPR160 protein expression. Data shown are means ± SEM; *P < 0.05 using unpaired *t* test. **k** Expression of the epithelial–mesenchymal transition (EMT) hallmarks E-cadherin, N-cadherin, and vimentin as well as GPR160 in blank and GPR160 gene transfected cells. **l** Quantitative densitometric analysis of **k**. **m** Expression of the three EMT hallmarks and GPR160 in blank and GPR160 gene silenced cells. **n** Quantitative densitometric analysis of **m**. **o** Expression of the EMT hallmarks ZO-1, snail, and fibronectin 1 in blank and GPR160 gene silenced cells. **p** Quantitative densitometric analysis of **o**. **q** Expression of the EMT hallmarks ZO-1, snail, and fibronectin 1 in blank and GPR160 gene transfected RWPE-1 cells. **r** Quantitative densitometric analysis of **q**. **s** Expression of the EMT hallmarks ZO-1, snail, and fibronectin 1 in blank and GPR160 gene transfected WPMY-1 cells. **t** Quantitative densitometric analysis of **s**. **u**, Migration of GPR160 gene silenced or transfected cells. **v** Quantitative densitometric analysis of **u**. **w** Invasion of GPR160 gene silenced or transfected cells. **x** Quantitative densitometric analysis of **w**. **y** Wound-healing assay in GPR160 gene silenced or transfected cells. Data presented are means ± SEM obtained from three independent experiments; *P < 0.05, **P < 0.01, and ***P < 0.001 using Student’s *t* test

As shown in Table S1, the mean age at diagnosis was 66.85 years, ranging from 51 to 82 years. Positive GPR160 RNA transcription was associated with higher Gleason grading scores (Fisher’s exact test, *P* = 0.026), the later stage of prostate cancer (Chi-square test, *P* = 0.045) and elevated PSA levels (unpaired *t* test, *P* = 0.048, between strong and moderate expressions, Figs. 1f–h). Table S2 illustrates the correlation between GPR160 protein levels and the clinical status of enrolled patients with the mean age at diagnosis of 66.84 years (ranging from 47 to 82 years). It was found that moderate GPR160 expression was linked with older ages compared with that of weak and strong expression groups (*P* = 0.009 and *P* = 0.026, respectively). GPR160 protein expression was also correlated with metastatic status using the M descriptor of the TNM (tumor, node, metastasis) classification system (*P* = 0.041, Figs. 1i–j).

The role of GPR160 in the metastatic potential of prostate cancer cell line 22Rv1 was investigated in parallel with the human prostate epithelial cell line RWPE-1 and human prostate stromal cell line WPMY-1. We employed the western blot technique to analyze GPR160 and the epithelial–mesenchymal transition (EMT) hallmarks including E-cadherin, N-cadherin, vimentin, snail, fibronectin 1, and ZO-1 in RWPE-1 and WPMY-1 cells following transfection of pCMV6-Entry vector encoding hGPR160. Figures 1k, 1l, 1q, and 1r show that increased GPR160 expression was associated with elevated E-cadherin and ZO-1 but decreased N-cadherin, vimentin, and snail levels in RWPE-1 cells. Figures 1s and 1t show that similar expression deference in WPMY-1 cells. To verify these results, we also detected the EMT hallmarks in GPR160-silenced prostate cancer cells (22Rv1). As displayed in Figs. 1m–p, the expression levels of E-cadherin and ZO-1 were decreased, whereas that of N-cadherin, vimentin, snail, and fibronectin 1 increased, suggesting that the GPR160 silenced cells (22Rv1) acquired invasive and metastatic properties, whereas RWPE-1 and WPMY-1 cells over-expressing GPR160 lost these properties. In phenotype assays, increased cell migration in GPR160 silenced cells (22Rv1 and PC3) and decreased cell migration in GPR160 overexpressed cells (RWPE-1 and WPMY-1) were observed (Figs. 1u and 1v). A similar trend was also noted in invasion assay (Figs. 1w and 1x). At the same time, wound healing assay exhibited more migration after silencing GPR160 in 22Rv1 and PC3 cells but less migration after over-expressing GPR160 in WPMY-1 cells (Fig. 1y).

As the second leading cause of death among men in the United States, poor prognosis is associated with advanced or metastatic prostate cancer. Despite the wide application of PSA in its diagnosis, novel and specific prostate biomarkers would certainly improve our ability to manage this deadly disease. In this study, we explored the relationship between GPR160 expression and the clinical status of prostate cancer. Both mRNA and protein levels of GPR160 were correlated with age, PSA, prostate size, Gleason score, TNM stage, lymph node metastasis, and nerve invasion in all enrolled patients.

Previous studies showed that the transcription level of GPR160 in several prostate cancer cell lines (PC3, LNCaP, DU145, and 22Rv1) was significantly higher and its expression was associated with apoptosis, microtubule cytoskeleton, cytokine activity, cell cycle, mitosis, and programmed cell death.^3^ The present study extended our scope to directly involve tissues from prostatectomy and to analyze GPR160 mRNA and protein levels in the context of clinical relevance. At the same time, both Gleason grade and stage classification are positively correlated with GPR160 mRNA levels (Table S1). This may provide an alternative parameter to predict the degree of malignancy in a semi-quantitative manner.

EMT is an important process during development by which epithelial cells acquire mesenchymal, fibroblast-like properties and show reduced intercellular adhesion and increased motility.^4^ It is implicated to play a critical role in tumor progression and malignant transformation by gaining invasive and metastatic properties. Alterations in EMT hallmarks such as decreases in E-cadherin and ZO-1 and increases in N-cadherin, vimentin, snail, and fibronectin 1 are indicative of cell mobility. Here, we provided evidence to link the presence of GPR160 to cell mobility according to the fact that most patients with positive GPR160 protein staining exhibited lower M scores.

In this study, there were 131 samples (53.7%) examined for GPR160 mRNA and protein in parallel. Among them, 5/36 (13.9%) of negative immunohistochemistry (IHC) samples were in agreement with that of fluorescence in situ hybridization (FISH), and 90/95 (94.8%) of IHC-positive samples were verified by that of FISH, giving a total consistency rate of 72.5% resulting in a Kappa coefficient of 0.111 (*P* = 0.097) and Fisher’s exact test *P* value of 0.137. A high discordance was observed among FISH+/IHC− patients (Table S3), suggesting that the level of GPR160 transcription does not correlate with that of translation. Discordant protein and mRNA expression have been well documented for gastric cancer, breast cancer and lung adenocarcinoma.^5^ In our case, the observed discordance might have been caused by a number of factors such as GPR160 translational regulation, different sensitivity of the two detection systems, receptor degradation by proteinase during assaying, etc. Obviously, post-transcriptional regulation of GPR160 in prostate cancer development requires in-depth investigation to further validate this biomarker.

GPCRs, as cell surface proteins to react with different ligands and transmit signals into intracellular molecules, have been implicated in the formation of various tumors. The involvement of GPR160 in prostate cancer demonstrated by this histological study, including correlation with the Gleason score, PSA level, metastasis, and prior treatment, offers potential application of this novel biomarker in targeted therapy and post-surgical evaluation of tumor malignancy and prognosis.

## Supplementary information

Supplemental Material

## Data Availability

The data sets used for the current study are available from the corresponding author upon reasonable request.
