# Supplementary material for: GPR160 is a potential biomarker associated with prostate cancer
Source: Signal Transduct Target Ther. 2021 Jun 25;6:241. doi: 10.1038/s41392-021-00583-7 (PMC8225807; doi:10.1038/s41392-021-00583-7)
Supplement: Supplementary file 1 — Supplemental Material [file 41392_2021_583_MOESM1_ESM.docx]

Supplementary Materials for

GPR160 is a potential biomarker associated with prostate cancer

Wanjing Guo, Junyu Zhang, Yan Zhou, Caihong Zhou, Yunjie Yang, Zhaotong Cong, Jibin Dong, Dehua Yang, Bo Dai and Ming-Wei Wang

Correspondence to: Ming-Wei Wang (mwwang@simm.ac.cn), Bo Dai (bodai1978@126.com) or Dehua Yang (dhyang@simm.ac.cn)

**This PDF file includes:**

Materials and Methods

Figure S1

Tables S1 to S3

Materials and Methods

Prostate tissue samples

Prostate tissue samples were obtained from patients underwent radical prostatectomy (RP) or transurethral resection of the prostate (TURP) at Shanghai Cancer Center, Fudan University. The institutional research ethics committee approved the present study and written informed consent forms were provided to all enrolled patients. Clinical information of each patient was gathered from the electronic record system showing that some of them had received anti-androgen therapy (*e.g*., flutamide or bicalutamide) prior to the surgery. Freshly collected prostate tissues were fixed in 4% formaldehyde upon receipt followed by paraffin-embedding for histological examination. A total of 224 patients with prostate cancer were enrolled from December, 2016 to March, 2019, including 211 RP and 13 TURP. Among them, 199 samples were performed with in situ hybridization (FISH), 158 samples with immunohistochemistry (IHC), and 131 patients were assessed by both methods, i.e., some patients were only assessed by one of the two histological parameters (Supplementary Fig. 1). Prostate tissue sections used for in situ hybridization and immunohistochemistry were prepared at Shanghai Ruiyuan Pharmatech Co., Ltd. and Shanghai Riqibio Co., Ltd., and clinical pathology observations were made at Shanghai Cancer Center, Fudan University.

In situ hybridization

GPR160 probe was designed and synthesized by Advanced Cell Diagnostics (Newark, CA, USA). The expression of GPR160 was detected using RNAscope^®^ 2.5 HD detection kit-RED ([NM_014373.2](http://www.ncbi.nlm.nih.gov/nuccore/NM_014373.2), region 433-1704, catalogue number 482681; Advanced Cell Diagnostics). After baking at 60°C, transverse tissue sections (4.5 μm thick) were deparaffinized in xylol twice (5 min each), absolute alcohol twice (1 min each) and dried at room temperature (RT). Hydrogen peroxide was applied to the sections drop-wise and kept at RT for 10 min. Sections were immersed in boiling RNAscope^®^ 1× Target Retrieval Regents for 8 min and dipped in water three times thereafter. RNAscope^®^ Protease Plus was added to the samples drop-wise followed by GPR160 probe for incubation at 40°C in a HybEZ™ oven for 2 h. After a series of standard signal amplification steps, tissues were stained with detection reagents (Fast RED-A and Fast RED-B), counterstained with 50% hematoxylin (Sigma-Aldrich, St. Louis, MO, USA), dehydrated and mounted with mounting medium (Vectormount, Burlingame, CA, USA).

Immunohistochemistry

Immunohistochemical staining was carried out on tissue samples that were deparaffinized in xylol and rehydrated using descending solutions of alcohol. The sections were reacted with 3% H_2_O_2_ to block endogenous [peroxidases](https://www.sciencedirect.com/topics/medicine-and-dentistry/peroxidase), washed by distilled water, immersed in antigen retrieval buffer at 98°C for 8 min and cooled at RT naturally. Each slide was then blocked by 5% BSA (Abcone, Shanghai, China) for 2 h at RT and incubated at 4°C overnight with anti-hGPR160 primary antibody (Biorbyt, Cambridge, UK) diluted 1:800 in PBS. The slides were washed with PBS three times and reacted with horseradish peroxidase (HRP)-conjugated anti-rabbit secondary antibody (Cell Signaling Technology, Danvers, MA, USA) diluted 1:5,000 in PBS for 30 min at RT. After rinsing with PBS, color was developed by DAB (Servicebio, Wuhan, China) for 1.5 min, and the sections were counterstained using 50% hematoxylin (Sigma-Aldrich), dehydrated and mounted with mounting medium (Vectormount).

GPR160 expression score

The level of GPR160 expression was analyzed semi-quantitatively according to the staining intensity of positive cells where dye sediment was concentrated. Expression score was determined to be 0 (negative), 1 (low), 2 (moderate) or 3 (high). 0 represents no staining or less than 1 dot in every 10 cells; 1 means 1–3 dots per cell without visible cluster; 2 denotes 4-7 dots per cell with few dot clusters; and 3 corresponds to >7 dots per cell with distinct dot clusters.

Cell culture and transfection

The human prostate epithelial cell line, RWPE-1, was purchased from American Tissue Culture Collection (Manassas, VA, USA) and cultured in keratinocyte serum free medium containing bovine pituitary extract (BPE, 0.05 mg/mL) and epidermal growth factor (EGF, 5 ng/mL) at 37°C with 5% CO_2_. The human prostate stromal cell line, WPMY-1, was purchased from the National Collection of Authenticated Cell Cultures (Shanghai, China) and cultured in Dulbecco's Modified Eagle's Medium (DMEM) containing fetal bovine serum (FBS, 5%). They were seeded in clear 12-well plates at a density of 3×10^5^ cells/mL or 2×10^5^ cells/mL and incubated for 24 h prior to transfection with pCMV6-Entry vector encoding hGPR160 (2 μg) using Lipofectamine 2000 reagent (5 μL/well) and Lipofectamine 3000 regent (3 μL/well, ThermoFisher Scientific, Carlsbad, CA, USA) suspended in 100 μL Opti-MEM (Invitrogen, Carlsbad, CA, USA) according to manufacturer’s protocol, followed by medium change 5 h later.

Western blot

RWPE-1, WPMY-1 and 22Rv1 cells were lysed with RIPA lysis buffer (Sigma-Aldrich) on ice for 5 min and centrifuged at 12,000 rpm for 15 min. Protein concentration of the supernatant was determined by BCA protein assay kit (Beyotime, Shanghai, China). Proteins were loaded onto 10% sodium dodecyl sulfate-polyacrylamide gel with 5× sodium dodecyl sulfate-loading buffer (Beyotime), separated by electrophoresis and transferred to polyvinylidene difluoride membranes (0.2 μm; Merck Millipore, Tullagreen, Ireland). The membranes were blocked with 5% fat free milk in TBST buffer for 2 h at RT and incubated overnight at 4°C with primary antibodies (1:1,000; anti-GPR160 rabbit polyclonal antibody from Biorbyt; anti-E-cadherin monoclonal antibody, anti-vimentin monoclonal antibody, and anti-fibronectin 1 monoclonal antibody from Absin, Shanghai, China; anti-N-cadherin monoclonal antibody from Abcam, Cambridge, UK; anti-α-Actin monoclonal antibody from Proteintech, Rosemont, IL, USA as well as anti-GAPDH monoclonal antibody, anti-snail monoclonal antibody and anti-ZO-1 monoclonal antibody from Cell Signaling Technology). After washing 3 times with TBST buffer, the membranes were incubated with secondary antibodies (1:5000; Cell Signaling Technology) for 1.5 h at RT. Protein bands were visualized by ECL Plus (Bio-Rad, Hercules, CA, USA). Densitometric analysis was then performed to determine the relative expression of target proteins normalized to GAPDH or α-Actin.

Lentivirus infection

HEK293T cells were transfected with corresponding pLVX-shRNA2 vectors, the packaging plasmids psPAX2 and pMD2.G (Addgene, Cambridge, MA, USA) using Lipofectamine 2000 reagents (ThermoFisher Scientific). The viral particles were harvested at 72 h after centrifugation. Human prostate cell lines 22Rv1 and PC3 that express high levels of GPR160 were infected (3×10^5^ cells/mL) with the viral particles (100 μL/well) in the presence of 6 µg/mL polybrene (Sigma) in 12-well plates. The silencing effect on GPR160 was detected by RT-PCR.

Transwell assay

For migration assay, 22Rv1 and PC3 silenced cells as well as WPMY-1 and RWPE-1 overexpression cells were seeded, respectively, into transwell units (Corning, NY, USA) containing 100 μL culture medium (RPMI1640, F-12K, DMEM or keratinocyte-serum-free medium, KSFM) with 1% FBS, and 600 μL of the same medium with 20% FBS were administered to the companion plate. For invasion assay, cells were seeded into transwell units containing 100 μL of the above medium without FBS, and 600 μL of which with 20% FBS were introduced to the companion plate. The plates were then incubated at 37°C with 5% CO_2_ for 24 h. Microscopic examination (Olympus, Tokyo, Japan) was performed to evaluate the efficiency.

Wound healing assay

Cells transfected with viral particles or pCMV6-Entry-GPR160 vector were seeded into 24-well plates. After the cell density reached 80% confluence, a wound was created by scratching the cell monolayer with a 100 µL pipette tip. Wound healing was monitored, and the migration distance was imaged at different time points. Mitomycin C (2 μg/mL) was added to the medium to reduce interference of cell proliferation.

Statistical analysis

Continuous variables are shown as means±SEM. Differences between mean values were evaluated by Mann-Whitney *U* test for comparison between two groups, and categorical data were presented as numbers and compared by the Fisher's exact test or Chi-square test. Kappa consistency test and Fisher's exact test were used to test the agreement or relativity between FISH and IHC scoring. Two-sided statistical analysis was performed with Prism 6 (GraphPad, San Diego, CA, USA), Kappa consistency test was conducted using SPSS Statistics version 22 (IBM Corporation, Armonk, NY, USA) and P<0.05 is considered statistically significant.


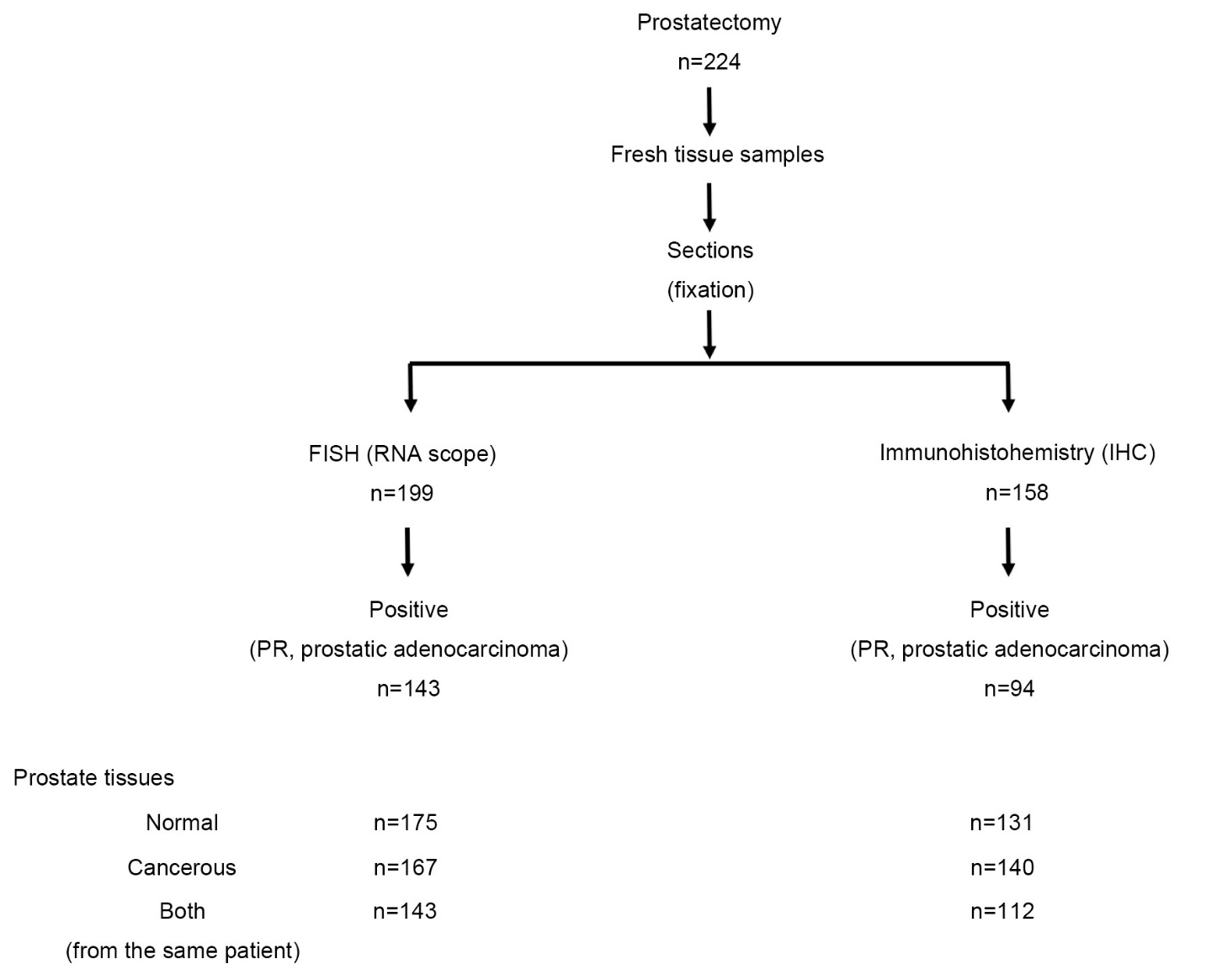


Figure S1. Workflow and number of cases assayed by FISH and IHC, respectively. PR, radical prostatectomy.

Table S1. Correlation between GPR160 mRNA levels and clinicopathological characteristics of enrolled patients.

|  | **Total** | **GPR160** | | **P value** | **GPR160** | | | **P value** | | |
| --- | --- | --- | --- | --- | --- | --- | --- | --- | --- | --- |
|  |  | **+** | **-** |  | **Low** | **Moderate** | **High** |  |  |  |
| **No. of patients** | 154 | 143 | 11 |  | 53 | 58 | 32 |  |  |  |
| **Age**  (Mean, range) | 66.85  (51-82) | 66.88  (51-82) | 66.45  (56-77) | 0.821 | 66.37  (51-82) | 67.45  (54-80) | 66.68  (52-80) | L *vs*. M 0.555 | M *vs.* H 0.643 | L *vs.* H 0.879 |
| **Gleason score** |  |  |  |  |  |  |  |  |  |  |
| ≤7 | 73 | 64 (44.8%) | 9  (81.8%) |  | 27 (50.9%) | 27  (46.5%) | 10 (31.3%) |  |  |  |
| ≥8 | 81 | 79 (55.2%) | 2  (18.2%) | 0.026* | 26 (49.1%) | 31  (53.5%) | 22 (68.7%) | 0.193 |  |  |
| **T** |  |  |  |  |  |  |  |  |  |  |
| T1+T2 | 71 | 65 (45.5%) | 6  (54.5%) |  | 26 (49.1%) | 27  (46.6%) | 12 (36.4%) |  |  |  |
| T3+T4 | 83 | 78 (54.5%) | 5  (45.5%) | 0.755 | 27 (50.9%) | 31  (53.4%) | 20 (63.7%) | 0.571 |  |  |
| **N** |  |  |  |  |  |  |  |  |  |  |
| N0 | 128 | 118 (82.5%) | 10 (90.9%) |  | 44 (83.0%) | 48  (82.8%) | 26 (81.3%) |  |  |  |
| ≥N1 | 26 | 25 (17.5%) | 1  (9.1%) | 0.692 | 9 (17.0%) | 10  (17.2%) | 6 (18.7%) | 0.977 |  |  |
| **M** |  |  |  |  |  |  |  |  |  |  |
| M0 | 137 | 128 (89.5%) | 9  (81.8%) |  | 46 (86.8%) | 55  (94.8%) | 27 (84.3%) |  |  |  |
| M1 | 17 | 15 (10.5%) | 2  (18.2%) | 0.348 | 7 (13.2%) | 3  (5.2%) | 5 (15.7%) | 0.216 |  |  |
| **Stage** |  |  |  |  |  |  |  |  |  |  |
| I | 2 | 2  (1.4%) | 0  (0.0%) |  | 2  (3.8%) | 0  (0.0%) | 0  (0.0%) |  |  |  |
| II | 33 | 27 (18.9%) | 6  (54.5%) |  | 10 (18.9%) | 12  (20.7%) | 5 (15.6%) |  |  |  |
| III | 86 | 83 (58.0%) | 3  (27.3%) |  | 31 (58.5%) | 33  (56.9%) | 19 (59.4%) |  |  |  |
| IV | 33 | 31 (21.7%) | 2  (18.2%) | 0.045* | 10 (18.8%) | 13  (22.4%) | 8 (25.0%) | 0.667 |  |  |
| **Nerve invasion** |  |  |  |  |  |  |  |  |  |  |
| Negative | 22 | 21 (14.7%) | 1  (9.1%) |  | 10 (18.9%) | 7  (12.1%) | 4 (12.1%) |  |  |  |
| Positive | 132 | 122 (85.3%) | 10 (90.9%) | 1.000 | 43 (81.1%) | 51  (87.9%) | 28 (87.9%) | 0.555 |  |  |
| **Lymph-vascular invasion** |  |  |  |  |  |  |  |  |  |  |
| Negative | 120 | 109 (76.2%) | 11 (100.0%) |  | 39 (73.6%) | 48  (82.7%) | 22 (68.7%) |  |  |  |
| Positive | 34 | 34 (23.8%) | 0  (0.0%) | 0.124 | 14 (26.4%) | 10  (17.3%) | 10 (31.3%) | 0..279 |  |  |
| **PSA**  (Mean, range, ng/mL/cm^3^) | 56.30  (1.05-1099) | 56.11  (1.05-1099) | 58.93  (4.07-485.3) | 0.151 | 52.46  (2.64-190.41) | 38.23  (1.05-206.6) | 95.13  (6.48-1099) | L *vs.* M 0.106 | M *vs.* H 0.048* | L *vs.*: H 0.508 |
| **Preoperative therapy** |  |  |  |  |  |  |  |  |  |  |
| None | 127 | 119 (83.2%) | 8  (72.7%) |  | 41 (77.4%) | 52  (89.7%) | 26 (81.3%) |  |  |  |
| ADT^a^ | 20 | 18 (12.6%) | 2  (18.2%) |  | 10 (18.9%) | 3  (5.2%) | 5 (15.6%) |  |  |  |
| ADT+Docetaxel^b^ | 7 | 6  (4.2%) | 1  (9.1%) | 0.631 | 2  (3.8%) | 3  (5.2%) | 1  (3.1%) | 0.268 |  |  |

*P<0.05 using Fisher's exact and Mann-Whitney *U* test for categorical and continuous variable analysis, respectively. L, low; M, moderate; and H, high.

^a^ADT, androgen deprivation therapy. ^b^Docetaxel, a drug used for chemotherapy.

Table S2. Correlation between GPR160 protein levels and clinicopathological characteristics of enrolled patients.

|  | **Total** | **GPR160** | | **P value** | **GPR160** | | | **P value** | | |
| --- | --- | --- | --- | --- | --- | --- | --- | --- | --- | --- |
|  |  | **+** | **-** |  | **Low** | **Moderate** | **High** |  |  |  |
| **No. of patients** | 128 | 94 | 34 |  | 55 | 30 | 9 |  |  |  |
| **Age**  (Mean, range) | 66.84  (47-82) | 66.71  (47-82) | 67.18  (54-77) | 0.828 | 65.54  (47-82) | 69.63  (54-80) | 64.11  (52-73) | L *vs*.  M 0.009** | M *vs.* H 0.026* | L *vs.* H 0.721 |
| **Gleason score** |  |  |  |  |  |  |  |  |  |  |
| ≤7 | 64 | 48 (51.1%) | 16 (47.1%) |  | 24 (56.4%) | 19  (63.3%) | 5 (55.5%) |  |  |  |
| ≥8 | 64 | 46 (48.9%) | 18 (52.9%) | 0.842 | 31 (43.6%) | 11  (36.7%) | 4 (44.5%) | 0.213 |  |  |
| **T** |  |  |  |  |  |  |  |  |  |  |
| T1+T2 | 62 | 45 (47.9%) | 17 (50.0%) |  | 24 (43.6%) | 15  (50.0%) | 6 (66.7%) |  |  |  |
| T3+T4 | 66 | 49 (52.1%) | 17 (50.0%) | 0.844 | 31 (56.4%) | 15  (50.0%) | 3 (33.3%) | 0.422 |  |  |
| **N** |  |  |  |  |  |  |  |  |  |  |
| N0 | 107 | 80 (85.1%) | 27 (79.4%) |  | 47 (85.5%) | 25  (83.3%) | 8 (88.9%) |  |  |  |
| ≥N1 | 21 | 14 (14.9%) | 7 (20.6%) | 0.431 | 8 (14.5%) | 5  (16.7%) | 1 (11.1%) | 0.913 |  |  |
| **M** |  |  |  |  |  |  |  |  |  |  |
| M0 | 115 | 88 (93.6%) | 27 (79.4%) |  | 52 (94.5%) | 28  (93.3%) | 8 (88.9%) |  |  |  |
| M1 | 13 | 6  (6.4%) | 7 (20.6%) | 0.041* | 3  (5.5%) | 2  (6.7%) | 1 (11.1%) | 0.811 |  |  |
| **Stage** |  |  |  |  |  |  |  |  |  |  |
| I+II | 33 | 23 (24.5%) | 10 (29.4%) |  | 12 (21.8%) | 8  (26.7%) | 3 (33.3%) |  |  |  |
| III | 71 | 57 (60.6%) | 14 (41.2%) |  | 36 (65.5%) | 17  (56.7%) | 4 (44.4%) |  |  |  |
| IV | 24 | 14 (14.9%) | 10 (29.4%) | 0.956 | 7 (12.7%) | 5  (16.6%) | 2 (22.3%) | 0.783 |  |  |
| **Nerve invasion** |  |  |  |  |  |  |  |  |  |  |
| Negative | 19 | 13 (13.8%) | 6 (17.6%) |  | 7 (12.7%) | 4  (13.3%) | 2 (22.2%) |  |  |  |
| Positive | 109 | 81 (86.2%) | 28 (82.4%) | 0.583 | 48 (87.3%) | 26  (86.7%) | 7 (77.8%) | 0.743 |  |  |
| **Lymph-vascular invasion** |  |  |  |  |  |  |  |  |  |  |
| Negative | 100 | 74 (78.7%) | 26 (76.5%) |  | 42 (76.4%) | 24  (80.0%) | 8 (88.9%) |  |  |  |
| Positive | 28 | 20 (21.3%) | 8 (23.5%) | 0.811 | 13 (23.6%) | 6  (20.0%) | 1 (11.1%) | 0.681 |  |  |
| **PSA**  (Mean, range, ng/mL/cm^3^) | 55.03  (2.64-1099) | 46.59  (2.64-183) | 78.55  (4.578-1099) | 0.328 | 48.55  (2.64-160.8) | 40.3  (6.0 –  183) | 55.13  (6.748-174.3) | L *vs*.  M 0.460 | M *vs*. H 0.914 | L *vs*. H 0.791 |
| **Treat** |  |  |  |  |  |  |  |  |  |  |
| Negative | 83 | 62 (66.0%) | 21 (61.8%) |  | 36 (65.5%) | 19  (63.3%) | 7 (77.8%) |  |  |  |
| Positive | 45 | 32 (34.0%) | 13 (38.2%) | 1.000 | 19 (34.5%) | 11  (36.7%) | 2 (22.2%) | 0.720 |  |  |
| **Preoperative therapy** |  |  |  |  |  |  |  |  |  |  |
| No | 107 | 78 (83.0%) | 29 (85.3%) |  | 45 (81.8%) | 26  (86.6%) | 7 (77.8%) |  |  |  |
| ADT^a^ | 16 | 12 (12.8%) | 4 (11.8%) |  | 8 (14.5%) | 2  (6.7%) | 2 (22.2%) |  |  |  |
| ADT+Docetaxel^b^ | 5 | 4  (4.2%) | 1  (2.9%) | 0.929 | 2  (3.7%) | 2  (6.7%) | 0  (0.0%) | 0.632 |  |  |

*P<0.05 and **P<0.01 using Fisher's exact and Mann-Whitney *U* test for categorical and continuous variable analysis, respectively. L, low; M, moderate; and H, high.

^a^ADT, androgen deprivation therapy. ^b^Docetaxel, a drug used for chemotherapy.

Table S3. Comparison of FISH and IHC results.

| **IHC result** | **GPR160 FISH negative** | **GPR160 FISH positive** | **Consistency with IHC** | **Inconsistency with IHC** |
| --- | --- | --- | --- | --- |
| Negative (n=36) | 5 | 31 | (5/36) 13.9% | (31/36) 86.1% |
| Positive (n=95) | 5 | 90 | (90/95) 94.8% | (5/95) 5.2% |
